# Cadherins Associate with Distinct Stem Cell-Related Transcription Factors to Coordinate the Maintenance of Stemness in Triple-Negative Breast Cancer

**DOI:** 10.1155/2017/5091541

**Published:** 2017-03-14

**Authors:** Chuanwei Yang, Xuemei Zhao, Naipeng Cui, Yulong Liang

**Affiliations:** ^1^Breast Medical Oncology, The University of Texas MD Anderson Cancer Center, Houston, TX, USA; ^2^College of Pharmacy, Taishan Medical University, Tai'an, Shandong, China; ^3^Department of Breast Surgery, Affiliated Hospital of Hebei University, Baoding, Hebei, China; ^4^The Michael E. DeBakey Department of Surgery, Baylor College of Medicine, Houston, TX, USA

## Abstract

Triple-negative breast cancer (TNBC) is an aggressive type of breast cancer with poor prognosis and is enriched in cancer stem cells (CSCs). However, it is not completely understood how the CSCs were maintained in TNBC. In this study, by analyzing The Cancer Genome Atlas (TCGA) provisional datasets and several small-size breast datasets, we found that cadherins (CDHs) 2, 4, 6, and 17 were frequently amplified/overexpressed in 47% of TNBC while E-cadherin (CDH1) was downregulated/mutated at 10%. The alterations of CDH2/4/6/17 were strongly associated with the elevated levels of several stem cell-related transcription factors (SC-TFs) including FOXM1, MCM2, WWTR1, SNAI1, and SOX9. CDH2/4/6/17-enriched genes including FOXM1 and MCM2 were also clustered and regulated by NFY (nuclear transcription factor Y) and/or EVI1/MECOM. Meanwhile, these SC-TFs including NFYA were upregulated in TNBC cells, but they were downregulated in luminal type of cells. Furthermore, small compounds might be predicted via the Connectivity Map analysis to target TNBC with the alterations of CDH2/4/6/17 and SC-TFs. Together with the important role of these SC-TFs in the stem cell regulation, our data provide novel insights into the maintenance of CSCs in TNBC and the discovery of these SC-TFs associated with the alterations of CDH2/4/6/17 has an implication in targeted therapy of TNBC.

## 1. Introduction

Breast cancer can be classified depending on the status of estrogen receptor (ER) and/or progesterone receptor (PGR) and epidermal growth factor receptor 2 (ERBB2/HER2) in clinic [1]. When all three markers ER, PGR and HER2 are negative in a tumor, it is called triple-negative breast cancer (TNBC). TNBC represents an aggressive type of breast cancer with poor prognosis [1]. The TNBC group is heterogeneous in nature, consisting of at least two major molecular subtypes including basal-like and claudin-low [2–4]. Both basal-like and claudin-low subtypes are enriched in cancer stem cells (CSCs) [4]. Although a lot of efforts have been made in this field, it is not fully understood how the stem cell population was enriched in TNBC.

Cadherins (CDHs) are a family of adhesion proteins consisting of more than 20 subtypes. These CDHs play important roles in cell-cell or cell-matrix junctions and regulate multiple aspects of fundamental cellular events such as cell polarity, motility, embryonic stem cell self-renewal and differentiation [5]. Among them, E-cadherin (CDH1) is a prototype and has been well characterized in stem cell maintenance and differentiation [6–8]. However, different subtypes of cadherins are expressed in distinct tissue types during development, and may involve in different aspect of cellular behavior. For instance, CDH1 is highly expressed in epithelial cells and modulate epithelial structure remodeling and the maintenance of epithelial stemness [6–8]. CDH2 (N-cadherin) is mostly expressed in the neuronal cells, and also involves in the process of epithelial-to-mesenchymal transition (EMT), which is correlated to the development of cancer stem cells (CSCs) [9–12]. Since CDH1 is critical for stem cell maintenance and regulation of epithelial cells, it raises a question whether other CDHs are involved in the maintenance of CSCs in breast cancer in addition to CDH1, especially under circumstances when CDH1 is deleted or lost, which remains relatively unexplored. In this study, we analyzed The Cancer Genome Atlas (TCGA) datasets containing over 1000 invasive breast cancer cases. We found that CDH2, 4, 6, and 17 were frequently amplified/overexpressed in breast cancer while CDH1 was downregulated/mutated. These changes affected the expression of several stem cell-related transcription factors (SC-TFs) such as NFYA and WWTR1, etc. Moreover, based on CDH2/4/6/17-enriched gene profiling, several small compounds might be predicted via the Connectivity Map (CMap) analysis to target TNBC with the alterations of CDH2/4/6/17 and SC-TFs. Altogether, our findings of these SC-TFs associated with the alterations of CDH2/4/6/17 may have an implication in targeted therapy of TNBC.

## 2. Materials and Methods

### 2.1. Breast Cancer Samples and Cell Lines Datasets

Breast cancer sample data used in this study were in whole or part based upon data generated by the TCGA Research Network (http://cancergenome.nih.gov/), which include the complete tumors group (*n* = 960) and triple-negative group (*n* = 116) from the Breast Invasive Carcinoma (TCGA provisional, *n* = 1105). TCGA datasets, as of December 15, 2016, contained the experimental data including gene mutations, copy number alterations (CNA), mRNA and protein expression and clinical data, and were retrieved from cBioPortal for Cancer Genomics (http://www.cbioportal.org/) [13]. Other breast cancer datasets used here including Gene Expression Omnibus (GEO) GSE3971 [14] and European Genome-phenome Archive (EGA) EGAS00000000083 [15] were accessed and retrieved from Oncomine (https://www.oncomine.org/). Breast cancer cell lines datasets (GEO GSE36139 and ArrayExpress E-MTAB-181) [16, 17] were retrieved from Cancer Cell Line Encyclopedia (CCLE) portal (http://www.broadinstitute.org/ccle) or UCSC Cancer Genomics Browser (http://xena.ucsc.edu/).

### 2.2. Data Analysis

Alterations of interested genes including amplifications, deletions, mutations, and up- or downregulation were retrieved from TCGA datasets with cBioPortal [13] or from other indicated datasets with Oncomine (https://www.oncomine.org/). Gene set enrichment analysis data (including mRNA level or RPPA [reverse phase protein assay]-based protein level) and survival data were also retrieved from cBioPortal with default parameters unless otherwise indicated. Gene Ontology (GO)/KEGG (Kyoto Encyclopedia of Genes and Genomes) or Reactome pathway analysis and transcription factor discovery were performed through the Database for Annotation, Visualization and Integrated Discovery (DAVID) Bioinformatics Resources 6.8 (https://david-d.ncifcrf.gov/). Heatmap was created from GenePattern according to the instructions (https://genepattern.broadinstitute.org/). Expression levels of stem cell-related transcription factors (SC-TFs) and several CDHs were retrieved from UCSC Cancer Browser with default parameters.

All CDH genes (*n* = 27) screened in this study include CDH1 (E-cadherin), CDH2 (N-cadherin), CDH3 (P-cadherin), CDH4 (R-cadherin), CDH5 (VE-cadherin), CDH6 (K-cadherin), CDH7 (cadherin 7), CDH8 (cadherin 8), CDH9 (T1-cadherin), CDH10 (T2-cadherin), CDH11 (OB-cadherin), CDH12 (N-cadherin 2), CDH13 (H-cadherin), CDH15 (M-cadherin), CDH16 (KSP-cadherin), CDH17 (LI-cadherin), CDH18 (cadherin 18), CDH20 (cadherin 20), PCDHGA12 (CDH21), CDH22 CDH23 CDH24 DCHS1 (CDH19, or CDH25), CDH26 DCHS2 (CDH27), CDHR3 (CDH28), and CDHR4 (CDH29).

SC-TFs used here include CTNNB1 (*β*-catenin), FOXM1, FOXO3, GLI2, HIF1A, HMGA1B, KLF4, MAF (c-MAF), MCM2, NANOG, POU5F1 (Oct-3/4), PRDM14, SNAI1 (Snail), SOX2, SOX9, STAT3, WWTR1, TBX3, TWIST1, ZEB1, LIN28A, LIN28B, and MYC (c-Myc) [18–30].

### 2.3. Flow Cytometry

Stem cell population of triple-negative (or claudin-low) breast cancer cells MDA-MB231 and luminal cells SKBR3 was determined by flow cytometry with stem cell marker CD24^−/low^CD44^+/high^. Fluorochrome-conjugated monoclonal antibodies against CD24 (FITC, Cat# 555478) and CD44 (PE, Cat# 555428) were purchased from BD Biosciences (San Diego, CA, USA). Fluorescent staining of CD24 and CD44 was performed as described elsewhere [31]. The labeled cells were finally analyzed on a FACS LSRII (BD Biosciences). The experiments were independently repeated.

### 2.4. Connectivity Map (CMap)

CDH2/4/6/17-enriched gene signature identified in this study, which included 101 genes with 12 downregulated and 89 upregulated, was used as a query for the CMap analysis according to the instructions (http://www.broadinstitute.org/cmap/) [32, 33]. CMap analyzes the association (i.e., the positive or negative correlation) between the given test signature and gene expression profiles of cell lines treated with specific concentrations of various drugs (perturbagens).

## 3. Results

### 3.1. CDH2/4/6/17 High/Amplified in Cooccurrence with CDH1 Low/Mutated Is Associated with TNBC

**Figure 1:**
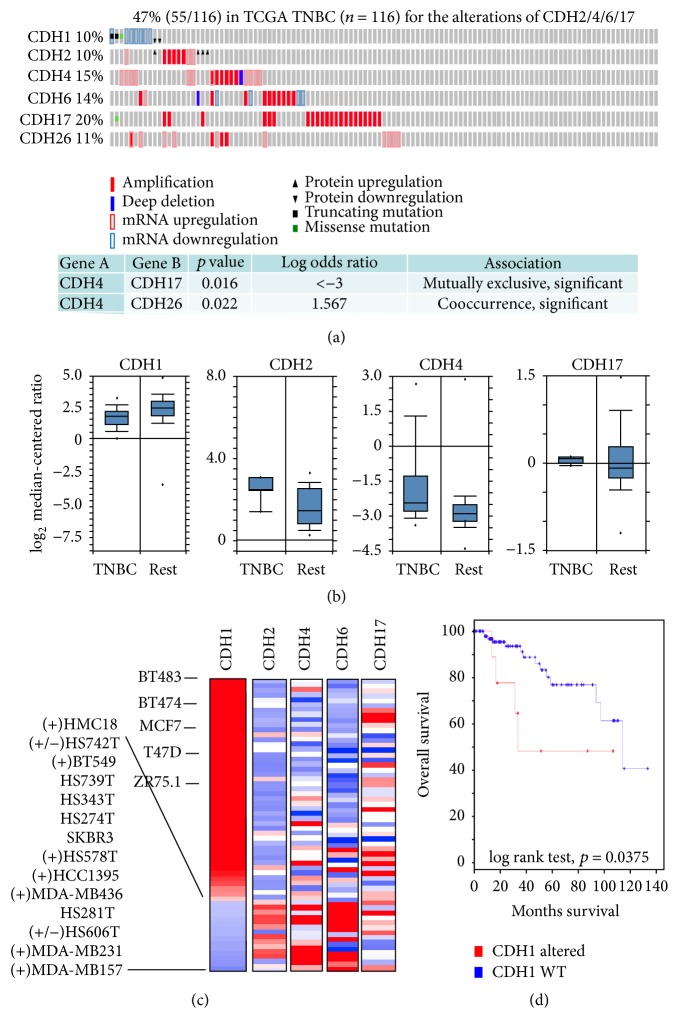
Alterations of CDHs in triple-negative breast cancer. (a) Alterations of CDH1, 2, 4, 6, 17, and 26 in TNBC samples from TCGA provisional datasets. The alterations here include deletion, amplification, downregulation, upregulation, and mutations. CDH1 was queried with EXP < −2.0 MUT HOMDEL PROT < −2.0 and other CDHs were queried with default parameters. Microarray data were used for mRNA expression level. The data were retrieved from cBioPortal as of December 15, 2016 (TNBC samples, *n* = 116). CDH, cadherin; TNBC, triple-negative breast cancer; TCGA, The Cancer Genome Atlas. EXP, mRNA expression level; MUT, mutation; HOMDEL, homozygous deletion; PROT, protein level as determined by reverse phase protein assay (RPPA). (b) Expression of CDH1, 2, 4, and 17 in TNBC compared to non-TNBC samples. These data were retrieved as of December 15, 2016, from Oncomine. Rest indicates non-TNBC samples. For CDH1 (from partial TCGA dataset), TNBC, *n* = 46; rest, *n* = 250. For CDH2 (from Curtis breast dataset, European Genome-phenome Archive accession number EGAS00000000083), TNBC, *n* = 4; rest, *n* = 17. For CDH4 (from partial TCGA dataset), TNBC, *n* = 49; rest, *n* = 300. For CDH17 (from Zhao breast dataset, GEO accession number GSE3971), TNBC, *n* = 5; rest, *n* = 37. (c) Expression of CDH1, 2, 4, 6, and 17 in CCLE breast cancer cell lines. The expression data (mRNA level) of CDH1, 2, 4, 6, and 17 were retrieved from breast cancer cell lines dataset (GEO accession number GSE36139) by suing CCLE portal according to the instructions. Red represents upregulation; blue means downregulation. The symbol (+) represents triple-negative breast cancer cells, and (+/−) for TNBC-like cells. CCLE, Cancer Cell Line Encyclopedia. (d) Overall survival of TNBC patients with or without CDH1 alterations. TNBC samples (*n* = 116) were obtained from TCGA provisional dataset and retrieved with cBioPortal. For CDH1 altered: *n* = 10 with median months survival = 33.97; for CDH1 WT: *n* = 106 with median months survival = 114.06.

As previously known, CDH1 is highly expressed in ductal invasive breast cancer, but low or absent in lobular invasive breast cancer [34, 35]. In terms of clinical classification based on ESR1/PGR or HER2 status, CDH1 expression is reduced in TNBC [36, 37]. Then the question arises what are the status of other CDHs in TNBC. Are they upregulated or downregulated or unchanged? Here, we wanted to determine whether the expression of other CDHs is altered in breast cancer, especially in TNBC since CDH1 is mostly downregulated or mutated in TNBC [36, 37]. To investigate the status of CDHs in breast cancer, we screened 27 members of CDHs in TCGA datasets and found that CDH1 was indeed low expressed or mutated in 16% of TCGA samples, and other CDHs (especially CDH17, CDH4, CDH26, CDH3/8, and CDH2/6/12) were almost highly expressed or amplified (Supplementary Figure  S1 in Supplementary Material at https://doi.org/10.1155/2017/5091541). In TNBC, CDH1, CDH2, CDH4, CDH6, CDH17, and CDH26 also exhibited marked alterations (Figure 1(a), Supplementary Figure  S2). Since CDH26 (11%) had a tendency to cooccur with CDH4 (15%) (Figure 1(a)), we herein selected CDH1/2/4/6/17 for further study. As for CDH1, our data showed that it had positive correlations with ESR1 or HER2 (Supplementary Table  S1), implying that CDH1 might be downregulated in TNBC (ESR1-/PGR- and HER2-). Actually, we found CDH1 was low expressed in TNBC than other breast cancers in a TCGA cohort (Figure 1(b)). However, CDH2/4/17 were remarkably altered in TNBC with high expression in most cases (Figure 1(b)). Also, we observed that CDH1 mRNA level was downregulated in most triple-negative cell lines from CCLE datasets (Figure 1(c)). Conversely, CDH2/4/6/17 (especially CDH2, 4, and 6) mRNA levels were aberrantly upregulated in these triple-negative cell lines (Figure 1(c)). Furthermore, CDH1 low was associated with poor overall survival of TNBC patients (Figure 1(d)). Therefore, these findings indicated that CDH2/4/6/17 high combined with CDH1 low might be of importance in TNBC.

### 3.2. Altered Expression of Stem Cell-Related Transcription Factors Is Concurrent with the Alterations of CDH2/4/6/17

Since CDH1 is vital for breast epithelial stem cell remodeling [5–7, 38–40], we wanted to answer the question if CDH2/4/6/17 are involved in breast CSCs under the condition of loss of CDH1. One way that the alterations of CDH2/4/6/17 influence the behavior of CSCs is through the changes in stem cell-related transcription factors (SC-TFs). Here, we selected 23 SC-TFs (listed in the Methods section) and screened their associations with the alterations of CDH2/4/6/17 in TCGA datasets via cBioPortal. As shown in Table 1, there were 5 SC-TFs including MCM2, WWTR1, FOXM1, SNAI1, and SOX9, having a tendency to be cooccurrent with the alterations of 3-4 CDHs of CDH2, 4, 6, and 17. Meanwhile, RPPA data confirmed that 4 SC-TFs (FOXM1, WWTR1, SNAI1, and MYC) were highly expressed with the alterations of CDH2/4/6/17 (Table 2); 3 out of these 4 SC-TFs belonged to the aforementioned 5 SC-TFs. In contrast, the alteration events (mutations or deletions) of CDH1 were mutually exclusive with high-level expressions of 9 out of 14 SC-TFs (Supplementary Tables  S2), suggesting that CDH1 might be not a key protein for the alterations of SC-TFs. Taken together, these findings indicated that the alterations of SC-TFs were cooccurring with the alteration events of CDH2/4/6/17.

### 3.3. CDH2/4/6/17-Associated Gene Enrichment Is Regulated by Stem Cell-Related Transcription Factors

**Figure 2:**
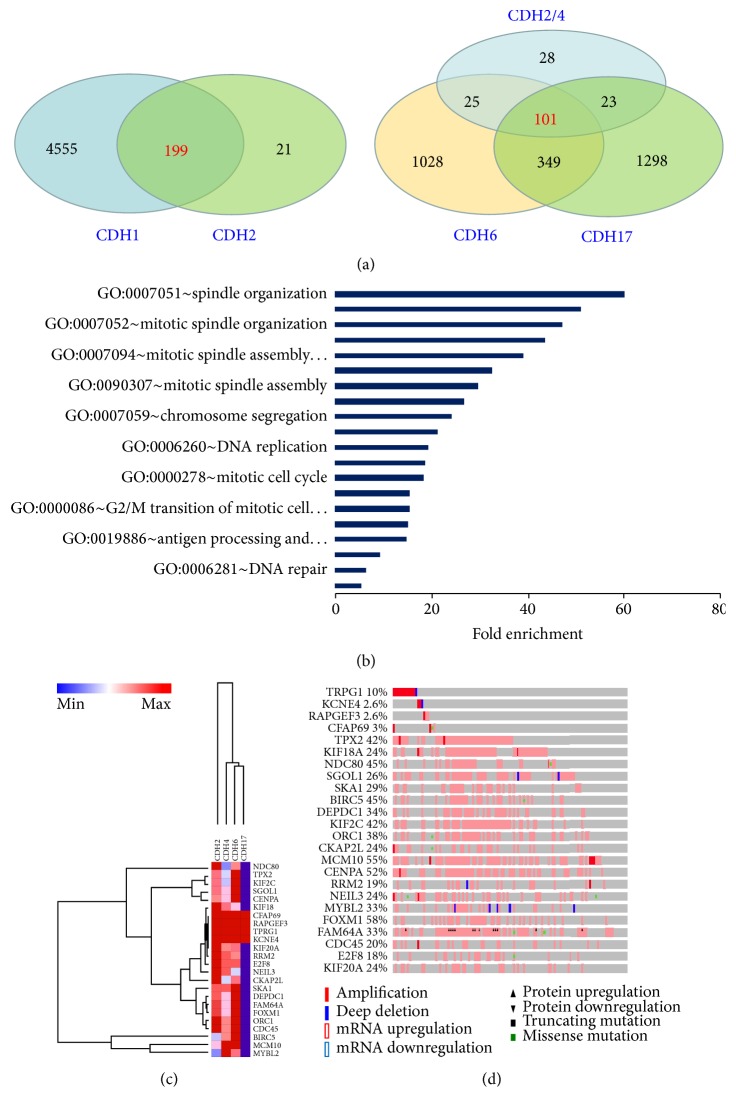
CDH2/4/6/17-enriched genes in breast cancer. (a) Venn diagrams showing the number of the overlaps between CDH1- and CDH2-enriched genes (left panel) and the overlaps between CDH2-, CDH4-, CDH6-, and CDH17-enriched genes (right panel). The enriched genes for each individual altered cadherin were retrieved with cBioPortal enrichments module from TCGA provisional breast datasets (complete tumor group, *n* = 960). CDH1 was queried with EXP < −2.0 MUT HOMDEL PROT < −2.0 and other CDHs were queried with default parameters. The genes were selected with *p* < 0.01 (derived from Student *t*-test) and *q* < 0.01 (derived from Benjamini-Hochberg procedure). EXP, mRNA expression level; MUT, mutation; HOMDEL, homozygous deletion; PROT, protein level as determined by reverse phase protein assay (RPPA). (b) Gene Ontology (GO) analysis of CDH2/4/6/17-enriched genes with DAVID Bioinformatics Resources 6.8. Fold enrichment indicates the magnitude of enrichment compared to population background regarding a given term. DAVID, Database for Annotation, Visualization, and Integrated Discovery. (c) Clustering analysis of top 24 out of 101 CDH2/4/6/17-enriched genes. Clustering analysis was performed in GenePattern according to the instructions. Min, minimal level; Max, maximal level. (d) Alterations of top 24 from 101 CDH2/4/6/17-enriched genes in TNBC. TNBC samples (*n* = 116) were obtained from TCGA provisional dataset as of December 15, 2016. The data were retrieved from cBioPortal with default parameters for mutations, copy number alterations, mRNA levels, and protein levels.

We next established CDH2/4/6/17-enriched gene signature to analyze whether the enriched genes can be regulated by SC-TFs. This is a different way to show the relationship between the alterations of CDH2/4/6/17 and SC-TFs. As shown in Figure 2(a), we first found that 199 genes were overlapped among CDH1- and CDH2-enriched genes; after CDH2-, CDH4-, CDH6-, and CDH17-enriched genes were considered together, we generated a refined list of CDH2/4/6/17-associated genes (101 genes) (Figure 2(a), Supplementary Table  S3), which were all contained in CDH1/2-enriched genes except 2 genes (KCNE4 and ZDHHC1). CDH2/4/6/17-associated genes were mainly involved in mitotic cell cycle and DNA replication/repair (Figure 2(b)). Further analysis showed that these genes possibly participated into several signaling pathways including the MAPK-ERK pathway and the PI3K-mTOR pathway (data not shown). Among the CDH2/4/6/17-associated 101 genes, top 24 genes were strongly correlated with the alterations of CDH2/4/6/17 (Figure 2(c)). Also, 20 out of these 24 genes were highly altered in TNBC (Figure 2(d), compared to Supplementary Figure  S3). Meanwhile, the aforementioned two transcription factors (MCM2 and FXOM1) were identified in this gene signature and highly expressed in the samples with the alterations of CDH2/4/6/17 (Supplementary Table  S3).

To further examine the effect of CDH2/4/6/17 on CSCs, we performed transcription factor discovery analysis by using DAVID Bioinformatics Resources 6.8. Interestingly, CDH2/4/6/17-enriched genes including two SC-TFs (MCM2 and FOXM1) were mainly grouped into a cluster that can be regulated by Nuclear Transcription Factor Y (NFY) and/or MECOM (EVI1) (Table 3), both of which have been reported to be involved in stem cell regulation [41, 42]. Therefore, our findings suggested that NFYs and MECOM might be the master transcription factors responsible for gene regulation and stem cell maintenance under the alterations of CDH2/4/6/17.

### 3.4. CDH2/4/6/17-Enriched Stem Cell-Related Transcription Factors Are Upregulated in TNBC

**Figure 3:**
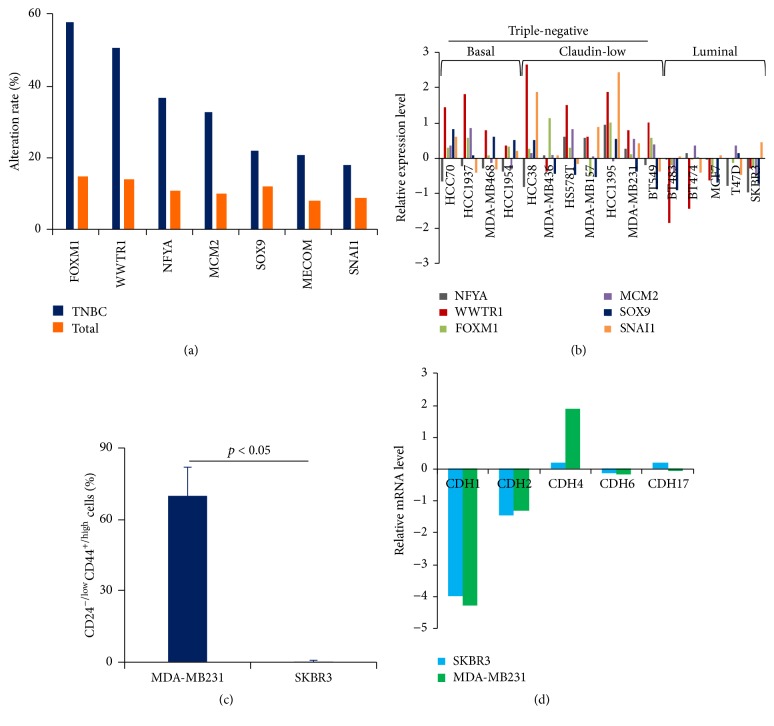
Stem cell-related transcription factors and cancer stem cells are enriched in TNBC cells. (a) The alteration rates of stem cell-related transcription factors (SC-TFs) in TCGA breast cancer samples (*n* = 960) or TCGA TNBC samples (*n* = 116). (b) The expression levels of SC-TFs were elevated in TNBC cells including basal-like and claudin-low. The expression data of the indicated SC-TFs were retrieved from breast cancer cell lines dataset (ArrayExpress accession number E-MTAB-181) by suing UCSC Cancer Genomic Browser according to the instructions. (c) The population of CSCs was higher in basal-like cells MDA-MB231 but lower in luminal subtype of cells SKBR3. The population of CSCs was determined by the staining of stem cell marker CD24^−/low^CD44^+/high^ followed by flow cytometry. (d) The expression levels of CDH1, 2, 4, 6, and 17 in TNBC claudin-low MDA-MB231 cells and luminal subtype of cells SKBR3. The expression data of CDH1, 2, 4, 6, and 17 in both cell lines were retrieved from the same dataset as described in (b).

SC-TFs play important roles in the maintenance of breast cancer stem cells as a whole. To gain insights into the changes of SC-TFs in clinical subtypes, especially in TNBC, we first analyzed the correlation of SC-TFs with clinical markers (ESR1, PGR, and HER2). We found that two SC-TFs such as FOXM1, MYC exhibited modest negative correlation with ESR1/PGR or HER2 status (Supplementary Table  S4). To further investigate the status of SC-TFs in TNBC, we analyzed the levels of CDH2/4/6/17-associated SC-TFs in the samples and cell lines of TNBC. All 7 SC-TFs (i.e., FOXM1, WWTR1, NFYA, MCM2, SOX9, MECOM, and SNAI1) were found to be amplified and/or upregulated in TCGA TNBC samples; the rates of their alterations were much higher than those in TCGA total breast samples (Figure 3(a) and Supplementary Figure  S4). Next, we retrieved the levels of 6 out of 7 SC-TFs (since no available data for MECOM) in breast cancer cell lines [17] from UCSC cancer genomics browser. Most of 6 SC-TFs were consistently upregulated in TNBC cell lines including basal-like and claudin-low (Figure 3(b)). Conversely, these SC-TFs were downregulated in luminal subtype of breast cancer cells (Figure 3(b)). In parallel, the proportion of cancer stem cell population determined by CD24^−/low^CD44^+/high^ was simultaneously higher in TNBC cell lines than other subtypes of breast cancer cell lines (Table 4). For instance, in claudin-low (TNBC) MDA-MB231 cells, there were 69.6% of them exhibiting CD24^−/low^CD44^+/high^, which was dramatically higher than luminal SKBR3 cells (0.21%) (Figure 3(c), Supplementary Figure  S5). Furthermore, at least CDH4 (among CDH2/4/6/17) was expressed at a higher level in MDA-MB231 than in SKBR3, while CDH1 was low expressed in both MDA-MB231 and SKBR3 (Figure 3(d)), as reported previously [43–45]. Therefore, these findings strongly suggested that CDH2/4/6/17-SC-TFs axis might play a key role in the enrichment of CSCs in TNBC.

### 3.5. TNBC Cells with the Alterations of CDH2/4/6/17-SC-TFs Axis May Be Targeted with the Perturbagens Discovered with the Connectivity Map

To evaluate whether TNBC cells with the alterations of CDH2/4/6/17-SC-TFs axis can be targeted, we adopted a web-based resource CMap. The CMap utilizes a pattern-matching algorithm to link the compounds (perturbagens) with physiological or pathological phenotypes by measuring similarities in gene expression, and therefore can be used to predict the affected pathways through the perturbagens affiliated with known molecular targets and signaling pathways, and discover potential pharmaceutical treatment based on the query signatures [32]. A CMap score from +1 to −1 is assigned based on the degree of similarity or dissimilarity between two signatures [32]. Here, we used the CDH2/4/6/17-associated signature to query the CMap. If a compound with a high CMap score (close to +1), it will have a similar gene pattern to that induced by CDH2/4/6/17 and may act on a parallel pathway induced by CDH2/4/6/17; if the score is close to −1, the perturbagen may counteract the effects induced by the alterations of these CDHs. First, we focused on the perturbagens, which are better related to known molecular targets and signaling pathways. For instance, we found that the CDH2/4/6/17-associated signature was similar to paclitaxel-induced signature (Table 5). Since paclitaxel is a microtubule-damaging agent and functions partially through p70S6K activation via multiple signaling pathways including ERK1/2 MAPK, JNK, PKC, Ca^++^, PI3K/mTOR [52], it provided evidence to support the finding revealed by analysis of GO/KEGG, as described above (Figure 2(b)). Next, we investigated whether some compounds can be used to target the cases with the CDH2/4/6/17-associated signatures. As shown in Table 6, multiple drugs were identified that had a significantly anticorrelating gene pattern to that induced by CDH2/4/6/17. For instance, resveratrol and its derivatives had been reported to exhibit anticancer activity in TNBC cells possibly through interfering with epigenetic regulation [53, 54]. Thus, our findings provided additional evidence to support the claim that the affected pathways were induced by CDH2/4/6/17 and revealed that the perturbagens predicted here might be used to target TNBC with the alterations of CDH2/4/6/17-SC-TFs axis.

## 4. Discussion

In this study, we evaluated the status of CDHs in TCGA breast cancer samples, especially in TNBC by using informatics and experimental analyses, and demonstrated that CDH2/4/6/17 themselves and their associated SC-TFs including WWTR1, NFYA, and FOXM1 might be involved in the enrichment of CSCs in TNBC.

CDH1 is an original cadherin and plays a pivotal role in epithelial structure remodeling and maintaining the stemness of stem cells in breast epithelial and cancer cells [5–7, 38–40]. However, CDH1 was low expressed in TNBC (Figure 1) [36, 37], and CDH1 alteration events were mutually exclusive with high-level alterations (amplification or upregulation) of most of SC-TFs used in this study in breast cancers (Supplementary Table  S2). As such, CDH1 may be not a key protein in the enrichment of CSCs in TNBC. Therefore, it raised a challenging question as to whether other CDHs have an effect on CSCs when CDH1 is low expressed or mutated in TNBC. Here, we shed lights on the CDHs and provided insights into the potential roles of CDH2, 4, 6, 17 in CSCs of TNBC. We clearly demonstrated that CDH2/4/6/17 alterations including amplifications and upregulation happened at a higher frequency (47%) in TNBC samples (Figure 1), which was correlated with the elevated expression of SC-TFs (Table 1), and also the enrichment of CSCs in TNBC (Figure 3 and Table 4), indicating CDH2/4/6/17 might have a potential role in the accumulation of CSCs in TNBC. As previously reported, CDH2 was often upregulated in cancers including breast cancers and acts as a promoting factor of cancer invasion and metastasis [10, 55, 56]. Moreover, CDH2 can regulate an EMT-like behavior [10], which is one of the properties belonging to the CSCs, and can even directly regulate stem cell fate decision [11, 12]. As for CDH4, CDH6, and CDH17, although there was rare emerging evidence to demonstrate their possible direct roles in the CSCs, a few reports demonstrated their functional roles in epithelial structure and even in regulation of EMT-like activity [57–60]. These studies are in consistency with our results and therefore support our notion here that CDH2, 4, 6, and 17 may have an implication in the CSCs in TNBC.

Our study here demonstrated that SC-TFs such as WWTR1, FOXM1, NFYA, and SOX9 might be involved in the enrichment of CSCs in TNBC (Figure 3, Tables 1 and 4). Consistently, these SC-TFs have been identified to be involved in stem cell regulation [22, 61–66]. For example, WWTR1 (also called TAZ), together with Yes-associated protein (YAP), is a key transcription regulator in Hippo-YAP signaling pathway, which is crucial for self-renewal of stem cells [28, 66–69]. Recently, several studies demonstrated that WWTR1/TAZ was strongly associated with triple-negative phenotype, and can sustain self-renewal potential of breast CSCs and increase the population of CSCs in TNBC [70–73]. When we prepared this manuscript, a report demonstrated that CDH2 indeed modulates mesenchymal cancer stem cells through WWTR1/TAZ [74], supporting our result of WWTR1's potential role in the enrichment of CSCs in TNBC induced by CDH2 in combination with CDH4, 6, and 17. Another important SC-TF is FOXM1. Likely, FOXM1 has also been reported to be involved in the EMT remodeling, self-renewal and the maintenance of stemness of stem cells [64, 75–77]. FOXM1 was highly upregulated in TNBC [78], confirming its role in the accumulation of CSCs in TNBC. Therefore, together with our results that CDH2/4/6/17 alterations were accompanied with elevated expression of SC-TFs (Tables 1 and 2), and TNBC are enriched in stem cell population (Figure 3, Table 4) [4, 79], this may provide a novel insight into the enrichment of CSCs in TNBC. In other words, CDH2/4/6/17 overexpressed individually or in combination might strengthen the inappropriate signals outside in, which may in turn trigger CDH2/4/6/17-related signal pathways to enhance the activity of SC-TFs such as WWTR1 and FOXM1, etc. The latter may then maintain the stem cell properties and increase the reservoir of CSCs in TNBC. Although this signal cascade remains to be investigated, our data provide a meaningful clue to further understand the mechanism underlying the maintenance of CSC in TNBC where CDH1 is often lost or mutated.

Although CDH1 is key for the maintenance of epithelial stem cells [5, 7, 80], many reports reveal that CDH1 loss is associated with EMT [81, 82], a cancer stem cell-like behavior [39], and its deficiency is characteristic of invasive breast lobular carcinomas, especially in TNBC [36, 37]. Our study showed that the level of CDH1 had a positive correlation with the status of ESR1 and HER2 (Supplementary Table  S1), and CDH1 was low expressed/mutated in TNBC (Figure 1). Together with our notion that CDH2/4/6/17 contributed to the enrichment of CSCs in TNBC, it raised a question that which part (CDH1 low or CDH2/4/6/17 high or in combination) was more important for the enrichment of CSCs in TNBC. Frankly speaking, it is still an open issue. However, our results here supported the hypothesis that the elevated level of CDH2/4/6/17 may have a prevailing effect on the accumulation of CSCs than the decreased level of CDH1 in TNBC. The high level of CDH-associated SC-TFs such as FOXM1, MCM2, SOX9, and SNAI1 were concurrent with the overexpression of CDH2, 4, 6, and 17 (Table 1), but mutually exclusive with the decrease of CDH1 (Supplementary Table  S2), strongly suggested that the enrichment of CSCs was more closely related to the high of CDH2, 4, 6, and 17, but not to the low of CDH1. Consistently, CDH2 has been identified as an indicator of EMT, and strongly correlated with CD133, one of the stem cell markers [11, 12, 83]. Moreover, CDH2 has a role in cell migration and metastasis over CDH1. For instance, CDH2 promotes cell motility in breast cancer cells regardless of the expression of CDH1 [84]. Also, CDH2 positivity in IHC staining was correlated to lymph node metastasis of TNBC, but CDH1 was not [85]. Altogether, our data support the hypothesis here that CDH2/4/6/17 may have a direct effect on the enrichment of CSCs in TNBC, which may have an implication in targeted therapy for TNBC in the future.

## 5. Conclusion

In this study, we demonstrated that CDH2/4/6/17 were amplified/overexpressed in TNBC, where the expression of CDH2/4/6/17-enriched SC-TFs was also increased. In parallel, these SC-TFs were strongly associated with the accumulation of CSCs in TNBC cells. Thus, we concluded that one mechanism through which the stemness is maintained in TNBC when E-cadherin is downregulated or lost is by way of upregulating the expression of other CDH family members such as CDH2/4/6/17 along with increased expression of SC-TFs. Moreover, we demonstrated that small compounds might be used to target TNBC based on alterations of CDH2/4/6/17 and SC-TFs. Although further studies have to be conducted on the mechanisms for regulation of SC-TFs by CDH2/4/6/17, all these observations highlight the potential functions of CDH2/4/6/17 on the reservoir of CSCs in TNBC with low expression or mutation of CDH1 and provide evidence with implications in targeted therapy in TNBC.

## Supplementary Material

Supplementary information contains 5 supplementary figures (Figure S1 to S5) and 4 supplementary tables (Table S1 to S4). Figure S1 and S2 show the alterations of all 27 CDHs in all breast cancer samples and TNBC samples from TCGA respectively. Figure S3 shows alteration of top 24 genes while Figure S4 demonstrates alterations of 7 stem cell-related transcription factors in breast cancer samples used for analysis. FigureS5 shows representative data of Figure 3C. Table S1 and S2 demonstrate the correlation of altered CDH1 and expressions of ESR1, PGR and HER2 or stem cell-related transcription factors respectively. Table S3 lists genes enriched with altered CDHs in breast cancer samples. Table S4 describes correlation between stem cell-related transcription factors and ESR1, PGR and HER2 status in breast cancer using RPPA dataset.

## Figures and Tables

**Table 1:** Cooccurrence of stem cell-related transcription factors in association with alterations of CDH2, 4, 6, and 17.

Gene A (CDH)^a^	Gene B (SC-TF)^b^	*p* value^c^	log odds ratio^d^	Association^e^
CDH2	**MCM2**	<0.001	1.340	Tendency for cooccurrence, significant
	**WWTR1**	<0.001	1.235	Tendency for cooccurrence, significant
	FOXO3	0.003	1.080	Tendency for cooccurrence, significant
	**FOXM1**	0.019	0.763	Tendency for cooccurrence, significant
	**SNAI1**	0.022	0.864	Tendency for cooccurrence, significant
	**SOX9**	0.099	0.553	Tendency for cooccurrence, marginal

CDH4	**FOXM1**	<0.001	1.256	Tendency for cooccurrence, significant
	**MCM2**	<0.001	1.181	Tendency for cooccurrence, significant
	**SNAI1**	<0.001	2.421	Tendency for cooccurrence, significant
	**SOX9**	<0.001	1.088	Tendency for cooccurrence, significant
	NANOG	0.004	1.281	Tendency for cooccurrence, significant
	POU5F1	0.004	0.949	Tendency for cooccurrence, significant
	HMGA1	0.005	0.854	Tendency for cooccurrence, significant
	MYC	0.017	0.494	Tendency for cooccurrence, significant
	**WWTR1**	0.057	0.472	Tendency for cooccurrence, marginal
	HIF1A	0.063	0.653	Tendency for cooccurrence, marginal

CDH6	GLI2	<0.001	1.532	Tendency for cooccurrence, significant
	**WWTR1**	<0.001	1.151	Tendency for cooccurrence, significant
	ZEB1	<0.001	1.737	Tendency for cooccurrence, significant
	**FOXM1**	0.005	0.827	Tendency for cooccurrence, significant
	KLF4	0.028	1.093	Tendency for cooccurrence, significant
	**SNAI1**	0.039	0.710	Tendency for cooccurrence, significant
	POU5F1	0.043	0.768	Tendency for cooccurrence, significant
	NANOG	0.062	0.968	Tendency for cooccurrence, marginal
	**MCM2**	0.071	0.593	Tendency for cooccurrence, marginal

CDH17	PRDM14	<0.001	2.551	Tendency for cooccurrence, significant
	LIN28B	<0.001	1.122	Tendency for cooccurrence, significant
	MYC	<0.001	>3	Tendency for cooccurrence, significant
	**FOXM1**	0.008	0.556	Tendency for cooccurrence, significant
	**SNAI1**	0.008	0.662	Tendency for cooccurrence, significant
	STAT3	0.009	0.692	Tendency for cooccurrence, significant
	**SOX9**	0.035	0.466	Tendency for cooccurrence, significant
	LIN28A	0.040	0.842	Tendency for cooccurrence, significant
	KLF4	0.041	−1.276	Tendency towards mutual exclusivity, significant
	GLI2	0.043	−0.842	Tendency towards mutual exclusivity, significant
	**MCM2**	0.048	0.460	Tendency for cooccurrence, significant
	TBX3	0.069	−0.693	Tendency towards mutual exclusivity, marginal
	FOXO3	0.073	0.418	Tendency for cooccurrence, marginal
	ZEB1	0.086	−0.653	Tendency towards mutual exclusivity, marginal

*Note*. ^a^CDH, cadherin: here including CDH2, 4, 6, and 17. ^b^SC-TF, stem cell-related transcription factor. SC-TFs in bold indicate that their occurrence simultaneously happened with the alterations of 3 or 4 CDHs mentioned in a. ^c^*p* values are derived from Fisher's exact test; *p* values less than 0.1 are included. ^d^log odds ratio indicates the likelihood that the events in genes A and B are mutually exclusive or cooccurrent across the selected cases. The value quantifies how strongly the presence or absence of alterations in gene A is associated with the presence or absence of alterations in gene B in the selected cases. ^e^log odds ratio > 0: association towards cooccurrence; log odds ratio ≤ 0: association towards mutual exclusivity; significant means *p* value < 0.05; marginal means *p* value is 0.05–0.1.

**Table 2:** Cooccurrence of SC-TFs with CDH2, 4, 6, and 17 by RPPA analysis^a^.

SC-TF	Locus	Expression change	*p* value^b^	*q* value^c^
FOXM1	12p13	Upregulation	1.98*E* − 07	6.69*E* − 06
SNAI1	20q13.2	Upregulation	2.15*E* − 04	1.24*E* − 03
WWTR1	3q23-q24	Upregulation	3.86*E* − 03	0.0136
MYC	8q24.21	Upregulation	0.0159	0.0417

*Note*. ^a^SC-TFs, stem cell-related transcription factors; CDH, cadherin; RPPA, reverse phase protein array. ^b^*p* value is derived from Student's *t*-test. *p* value < 0.05 indicates significant. ^c^*q* value is derived from Benjamini-Hochberg procedure. *q* value < 0.05 indicates significant.

**Table 3:** Potential transcription factors predicted using DAVID analysis^a^.

Transcription factor^b^	%^c^	Fold enrichment^d^	Statistics	
			*p* value^e^	Benjamini^f^
**NFY**	**61.63**	**1.520018153**	9.59**E** − 05	**0.016736215**
**EVI1**	**86.05**	**1.216541909**	8.52**E** − 04	0.072283654^*∗*^
E2F	63.95	1.335510686	0.002616935	0.142494828
MEF2	75.58	1.192911873	0.013046475	0.438881091
MEIS1	50.00	1.305125299	0.023933933	0.573746109
COMP1	52.33	1.270475168	0.031160597	0.604888864

*Note*. ^a^DAVID, Database for Annotation, Visualization, and Integrated Discovery version 6.8. ^b^Transcription factors in bold indicate significantly predicted transcription factors. EVI1, also called MECOM. ^c^% means the percentage of genes regulated by the indicated transcription factor among the total query genes. ^d^Fold enrichment indicates the magnitude of enrichment compared to population background regarding a given term. ^e^*p* value is derived from modified Fisher's exact test (also called EASE score). ^f^Benjamini indicates a more conservative method to control family-wide false discovery under certain rate. ^*∗*^Marginal significance.

**Table 4:** Cancer stem cell population in different breast cancer cell lines^a^.

Breast cancer cell lines	Stem cell markers^b^		
	CD24^−/low^CD44^+/high^ (%)	ALDH1 activity	Side population
*Basal-like/claudin-low*			
MDA-MB157	97.4% [46]^c^	14.0 ± 1.8% [47]	
HS578T	65% [48], 85 ± 5% [31], 99.3% [49]		
MDA-MB231	99.9% [46], 85 ± 5 [31], 98.6% [49], most cells [50]	13.0 ± 1.4% [47]	3.40 ± 0.60% [50]
MDA-MB436	72 ± 5% [31]		
HCC1937	17.9% [49]		
BT549	90.3% [46], 16.5% [49]		
*Luminal subtype*			
MCF-7	0% [31, 46], 0.028% [49], 1% [51]		
MDA-MB453	0% [46]		
T47D	0% [31, 46]		
SKBR3	0% [31, 46]		

*Note*. ^a^The data here are retrieved from the literature. ^b^CD24^−/low^CD44^+/high^ is a more powerful stem cell marker. ALDH1, aldehyde dehydrogenase 1. ^c^The numbers in parentheses are the reference numbers.

**Table 5:** Top 10 perturbagens identified through the Connectivity Map that induce the CDH-associated signature.

Pharmaceutical perturbagen	Enrichment CMap score	Rank^a^	*n* ^b^	*p* value	Description
Trimethobenzamide	0.885	5	5	0.00006	An antiemetic used to prevent nausea and vomiting
Felbinac	0.892	7	4	0.00012	A nonsteroidal anti-inflammatory drug of the arylacetic acid class
Iopamidol	0.886	8	4	0.00018	A radiopaque contrast agent
Diethylstilbestrol	0.738	13	6	0.00085	A synthetic, nonsteroidal estrogen, and a potent agonist of estrogen receptors
Adiphenine	0.789	14	5	0.00092	An inhibitor of nicotinic receptors
Paclitaxel	0.728	17	6	0.00107	A microtubule-damaging agent, affecting mitosis
Thioperamide	0.754	20	5	0.00216	A potent antagonist of histamine receptor H3/H4
Cinchonine	0.813	21	4	0.00237	A multidrug resistance-reversing agent
Diphenhydramine	0.737	25	5	0.00290	An antihistamine agent
Vinburnine	0.796	28	4	0.00334	A vinca alkaloid acting as a vasodilator

*Note*. ^a^Rank by *p* value. ^b^*n* indicates the number of instances related to this perturbagen tested in the Connectivity Map.

**Table 6:** Top 10 perturbagens identified through the Connectivity Map that anticorrelated with the CDH-associated signature.

Pharmaceutical perturbagen	Enrichment CMap score	Rank^a^	*n* ^b^	*p* value	Description^c^
Resveratrol	−0.765	1	9	<0.00001	A stilbenoid, a type of natural phenol
Trifluoperazine	−0.553	2	16	<0.00001	A blocker of dopamine D1/D2 receptor
0297417-0002B	−0.981	3	3	0.00004	N.D.
MG-262	−0.939	9	3	0.00032	A proteasome inhibitor
Apigenin	−0.866	10	4	0.00062	A potent inhibitor of CYP2C9; a monoamine transporter activator; a ligand for central benzodiazepine receptors
Pyrvinium	−0.740	11	6	0.00066	An antinematodal agent
Methotrexate	−0.654	12	8	0.00078	An inhibitor of dihydrofolate reductase (DHFR), participating in DNA repair; a suppressant of immunology
Amiodarone	−0.777	15	5	0.00096	A calcium channel blocker
Piperidolate	−0.908	19	3	0.00142	An antimuscarinic
Acepromazine	−0.811	24	4	0.00251	A phenothiazine derivative antipsychotic drug

*Note*. ^a^Rank by *p* value. ^b^*n* indicates the number of instances related to these perturbagens tested in the Connectivity Map. ^c^N.D., not determined.
