# Supplementary material for: Cadherins Associate with Distinct Stem Cell-Related Transcription Factors to Coordinate the Maintenance of Stemness in Triple-Negative Breast Cancer
Source: Stem Cells Int. 2017 Mar 14;2017:5091541. doi: 10.1155/2017/5091541 (PMC5368378; doi:10.1155/2017/5091541)
Supplement: Supplementary file 1 — Supplementary information contains 5 supplementary figures (Figure S1 to S5) and 4 supplementary tables (Table S1 to S4). Figure S1 and S2 show the alterations of all 27 CDHs in all breast cancer samples and TNBC samples from TCGA respectively. Figure S3 shows alteration of top 24 genes while Figure S4 demonstrates alterations of 7 stem cell-related transcription factors in breast cancer samples used for analysis. FigureS5 shows representative data of Figure 3C. Table S1 and S2 demonstrate the correlation of altered CDH1 and expressions of ESR1, PGR and HER2 or stem cell-related transcription factors respectively. Table S3 lists genes enriched with altered CDHs in breast cancer samples. Table S4 describes correlation between stem cell-related transcription factors and ESR1, PGR and HER2 status in breast cancer using RPPA dataset. [file 5091541.f1.pdf]

Cadherins associate with distinct stem cell-related transcription factors to coordinate the maintenance of stemness in triple negative breast cancer

Chuanwei Yang,[1] Xuemei Zhao,[2] Naipeng Cui, [1,3] and Yulong Liang [2,4] *

[1] Breast Medical Oncology, The University of Texas MD Anderson Cancer Center, Houston, TX, USA.
[2] College of Pharmacy, Taishan Medical University, Tai'an, Shandong, China.
[3] Department of Breast Surgery, Affiliated Hospital of Hebei University, Baoding, Hebei, China.
[4] The Michael E. DeBakey Department of Surgery, Baylor College of Medicine, Houston, TX, USA.
* Correspondence: Yulong Liang, Ph.D., Email: yulong@bcm.edu, or yulong.liang@yahoo.com.

SUPPLEMENTARY INFORMATION

Supplementary Figure S1: Alterations of all 27 CDHs in TCGA breast cancer datasets (n=960).

Supplementary Figure S2: Alterations of all 27 CDHs in TNBC samples from TCGA breast cancer datasets (n=116).

Supplementary Figure S3: Alterations of top 24 genes in TCGA breast cancer datasets (n=960).

Supplementary Figure S4: Alterations of 7 stem cell-related transcription factors in TCGA TNBC (n=116) (A) and in total TCGA breast samples (n=960) (B).

Supplementary Figure S5: Representative data of Fig. 3C.

Supplementary Table S1: Correlation of the alterations of CDH1 with ESR1, PGR, and HER2 status.

Supplementary Table S2: Correlation of the alterations of CDH1 with stem cell-related transcription factors.

Supplementary Table S3: List of genes enriched with the alterations of CDH2/4/6/17 in TCGA breast cancer datasets.

Supplementary Table S4: Correlation between stem cell-related transcription factors and ESR1, PGR, HER2 status.

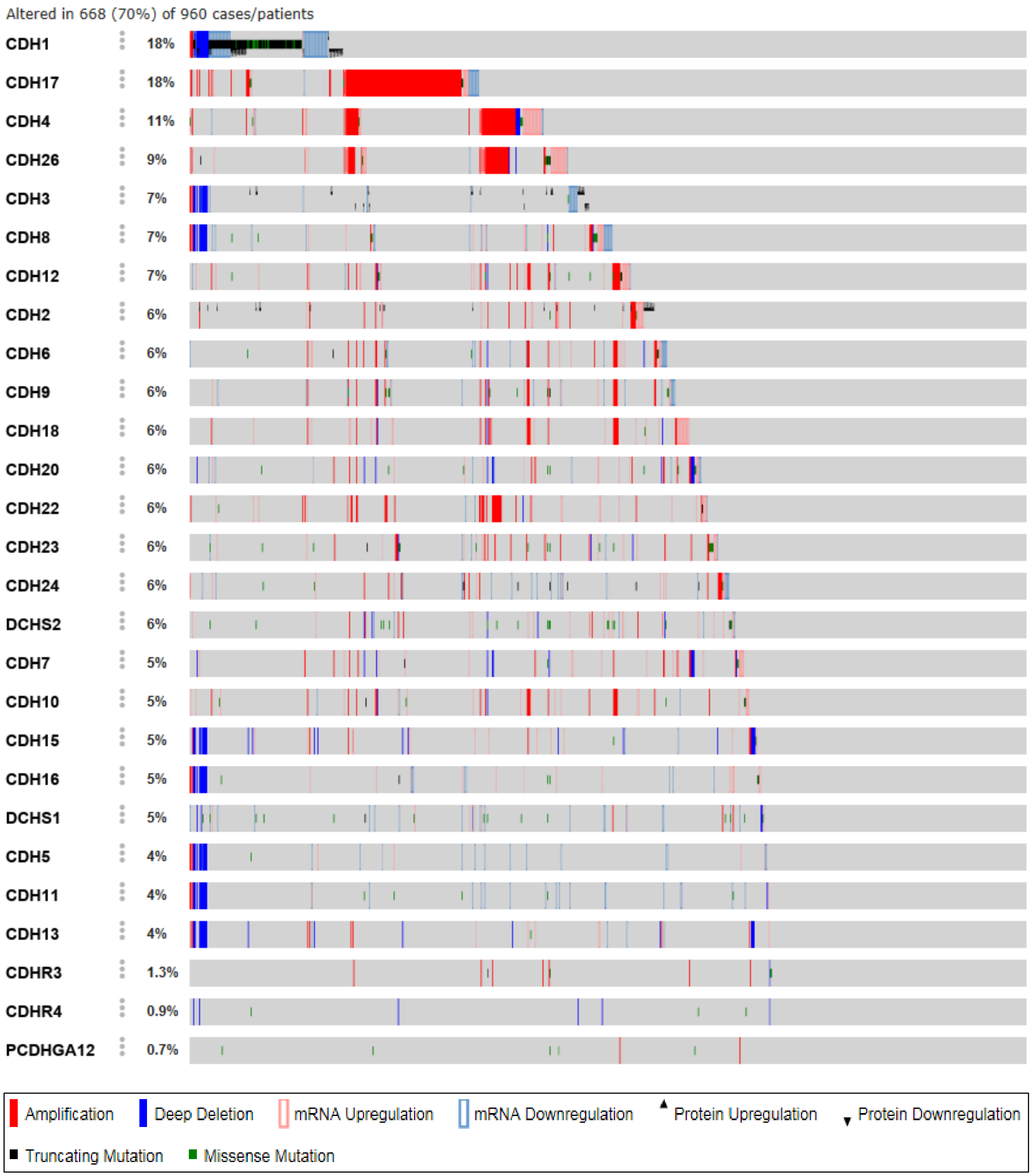

Figure S1: Alterations of all 27 CDHs in TCGA breast cancer datasets (n=960). These data were retrieved from cBioPortal with default parameters.

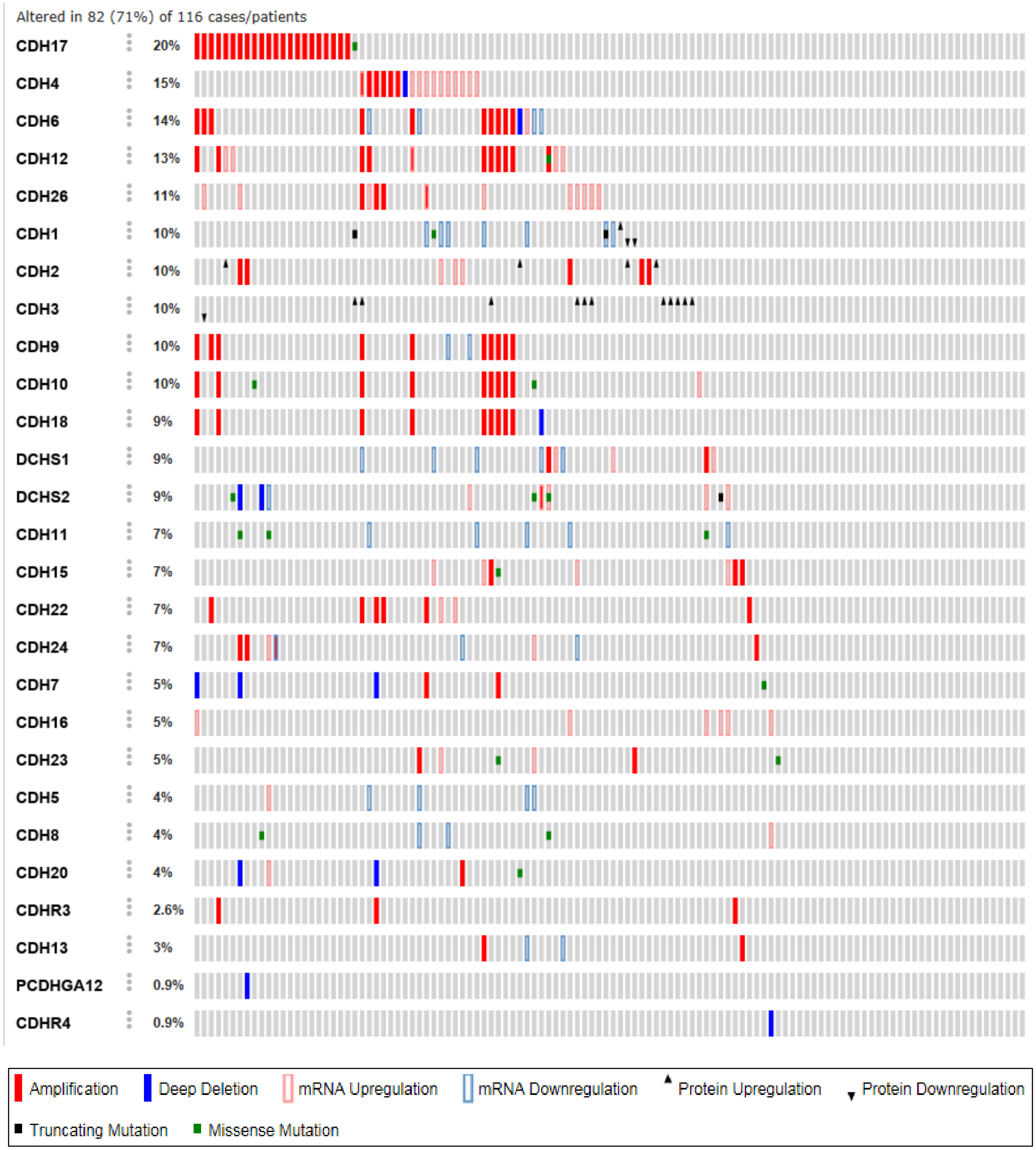

Figure S2: Alterations of all 27 CDHs in TNBC samples from TCGA breast cancer datasets (n=116). These data were retrieved from cBioPortal with default parameters.

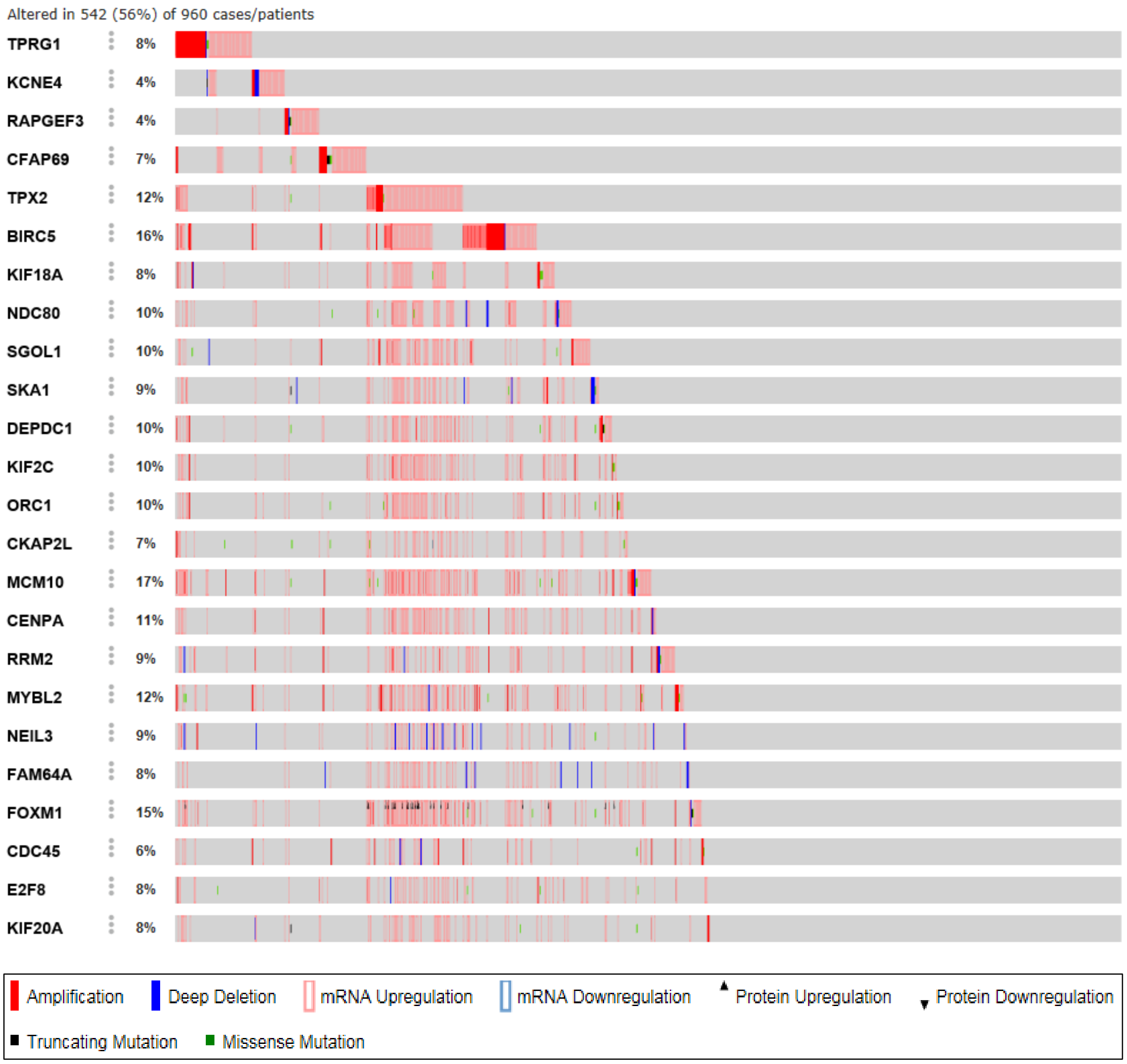

Figure S3: Alterations of top 24 genes in TCGA breast cancer datasets (n=960). These data were retrieved from cBioPortal with default parameters.

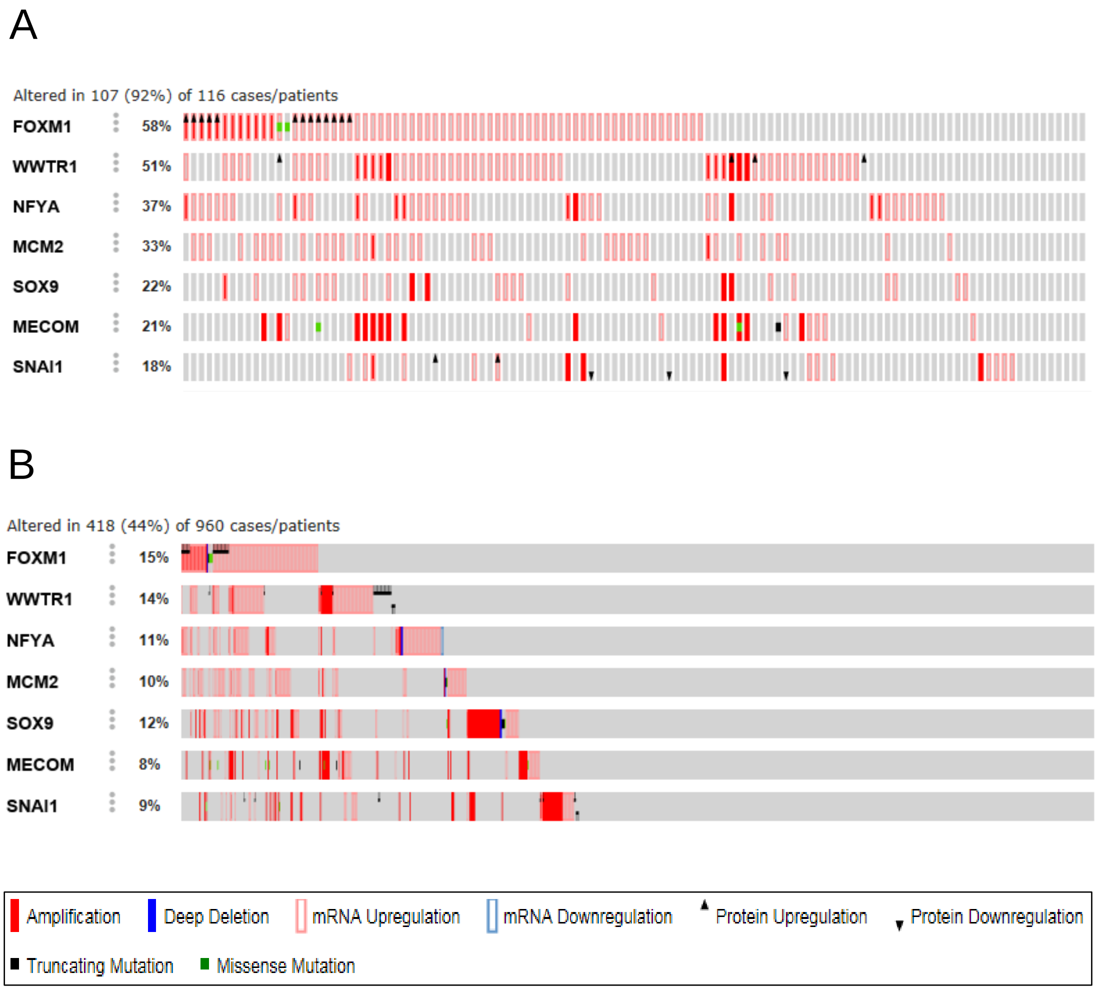

Figure S4: Alterations of 7 stem cell-related transcription factors in TCGA TNBC (n=116) (A) and in total TCGA breast samples (n=960) (B).

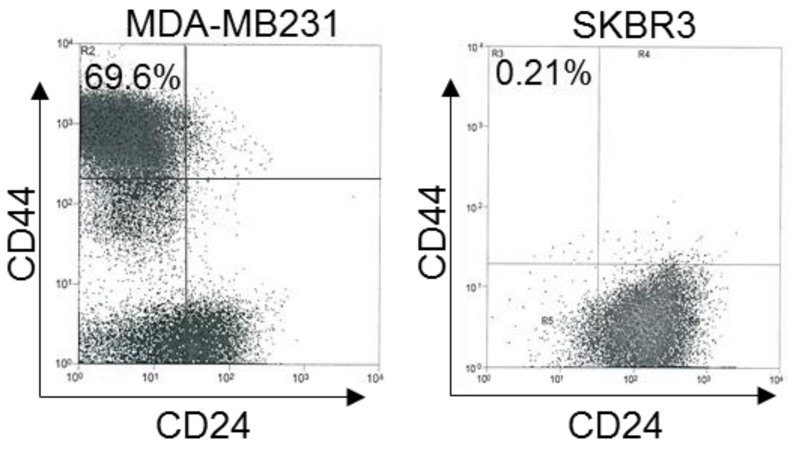

Figure S5: Representative data of Fig. 3C.

Table S1. Correlation of the alterations of CDH1 with ESR1, PGR, and HER2 status.

Protein A [a]	Protein B [b]	Correlation [c]	p value [d]
CDH1	ESR1	0.2639	2.1627e-15
CDH1	PGR	0.0065826	0.84592
CDH1	HER2	0.3075	1.3521e-20

Note: Data analysis was done on a dataset of TCPA Breast Invasive Carcinoma with 901 samples where RPPA data were available (http://tcpaportal.org/tcpa).
a, CDH1, E-cadherin; *b*, ESR1, estrogen receptor α; PGR, progesterone receptor; HER2, epidermal growth factor receptor 2 (ERBB2); *c*, Correlation analysis is based on Spearman's rank test; *d*, p value <0.05 indicates significant.

Table S2. Correlation of the alterations of CDH1 with stem cell-related transcription factors.

Gene A	Gene B (SC-TF) [a]	p value [b]	Log odds ratio [c]	Association [d]
CDH1	**FOXM1**	<0.001	-1.074	Tendency towards mutual exclusivity, Significant
	**HMGA1**	<0.001	-1.327	Tendency towards mutual exclusivity, Significant
	**MCM2**	<0.001	-1.506	Tendency towards mutual exclusivity, Significant
	TBX3	<0.001	1.052	Tendency for co-occurrence, Significant
	MAF	0.004	0.968	Tendency for co-occurrence, Significant
	**POU5F1**	0.008	-1.085	Tendency towards mutual exclusivity, Significant
	**HIF1A**	0.016	-1.093	Tendency towards mutual exclusivity, Significant
	GLI2	0.017	0.708	Tendency for co-occurrence, Significant
	**SOX9**	0.027	-0.605	Tendency towards mutual exclusivity, Significant
	KLF4	0.032	0.792	Tendency for co-occurrence, Significant
	CTNNB1	0.041	0.561	Tendency for co-occurrence, Significant
	**SNAI1**	0.047	-0.603	Tendency towards mutual exclusivity, Significant
	**NANOG**	0.057	-1.199	Tendency towards mutual exclusivity, Marginal
	**PRDM14**	0.087	-0.382	Tendency towards mutual exclusivity, Marginal

Note: *a*, SC-TF, stem cell-related transcription factor. SC-TFs in bold indicate those having mutual exclusivity with the alterations of CDH1.

b, p values are derived from Fisher's Exact Test; p values less than 0.1 are included.

c, Log odds ratio indicates the likelihood that the events in genes A and B are mutually exclusive or co-occurrent across the selected cases.

d, Log odds ratio > 0: Association towards co-occurrence; Log odds ratio <= 0: Association towards mutual exclusivity; Significant means p value < 0.05; marginal means p value is 0.05-0.1.

Table S3: List of genes enriched with the alterations of CDH2/4/6/17 in TCGA breast cancer datasets.

PROBE	SYMBOL	CDH2	CDH4	CDH6	CDH17
ILMN_1790350	TPRG1	-1.59	-1.38	-1.45	-1.01
ILMN_1703110	KCNE4	-1.19	-0.76	-1.38	-0.94
ILMN_1725099	RAPGEF3	-0.89	-0.59	-0.93	-0.36
ILMN_1790315	CFAP69	-0.81	-0.6	-0.88	-0.51
ILMN_101903	APH1B	-0.74	-0.39	-0.75	-0.5
ILMN_1728581	C3ORF18	-0.72	-0.53	-0.52	-0.34
ILMN_164548	DLG4	-0.61	-0.45	-0.43	-0.27
ILMN_2094313	ZDHHC1	-0.56	-0.41	-0.42	-0.31
ILMN_1787718	SLC27A1	-0.55	-0.31	-0.59	-0.22
ILMN_5011	ZBTB4	-0.36	-0.34	-0.38	-0.23
ILMN_1774427	CALCOCO1	-0.28	-0.32	-0.46	-0.18
ILMN_15629	RBM5	-0.27	-0.21	-0.33	-0.18
ILMN_1706246	CCT5	0.39	0.43	0.61	0.3
ILMN_28740	SSRP1	0.4	0.29	0.41	0.14
ILMN_1778717	DBF4	0.44	0.47	0.47	0.25
ILMN_2405521	MTHFD2	0.46	0.32	0.4	0.28
ILMN_1727055	PARPBP	0.51	0.52	0.56	0.36
ILMN_1724407	TACC3	0.51	0.54	0.56	0.39
ILMN_2160929	FEN1	0.53	0.47	0.45	0.23
ILMN_1708160	KPNA2	0.54	0.52	0.74	0.46
ILMN_915	SPC25	0.55	0.61	0.53	0.39
ILMN_1720526	CENPN	0.56	0.76	0.7	0.34
ILMN_2363027	RAD51	0.57	0.67	0.56	0.49
ILMN_4098	CDKN3	0.58	0.61	0.6	0.46
ILMN_2053650	AURKA	0.59	1.1	0.75	0.66
ILMN_1664630	CHEK1	0.6	0.5	0.67	0.31
ILMN_22512	PRC1	0.6	0.66	0.73	0.4
ILMN_1787280	AUNIP	0.61	0.83	0.66	0.48
ILMN_1710428	CDK1	0.62	0.7	0.58	0.48
ILMN_2413650	STIL	0.62	0.58	0.71	0.36
ILMN_2112460	MAD2L1	0.65	0.69	0.69	0.48
ILMN_183916	MCM2	0.65	0.59	0.49	0.34
ILMN_182117	MKI67	0.65	0.67	0.74	0.51
ILMN_1664533	NUF2	0.66	0.72	0.67	0.51
ILMN_1697751	GINS1	0.67	0.63	0.54	0.47
ILMN_1796589	TRIP13	0.68	0.73	1.05	0.54
ILMN_138188	ARHGAP11A	0.69	0.57	0.6	0.39
ILMN_1797307	BUB1B	0.69	0.56	0.56	0.37
ILMN_869	SKA3	0.69	0.69	0.65	0.5
ILMN_8503	KIF11	0.7	0.55	0.52	0.38
ILMN_1278	RAD54L	0.7	0.76	0.88	0.5
ILMN_1674662	TICRR	0.7	0.98	1.06	0.53
ILMN_11802	KIF23	0.71	0.67	0.67	0.43

ILMN_1751444	NCAPG	0.71	0.78	0.64	0.56
ILMN_1736176	PLK1	0.71	0.78	0.92	0.64
ILMN_177161	CCNB2	0.72	0.81	0.85	0.55
ILMN_18763	CDCA3	0.72	0.74	0.84	0.63
ILMN_1709085	GSG2	0.73	0.56	0.81	0.38
ILMN_2126706	LMNB1	0.73	0.7	0.49	0.37
ILMN_25893	PTTG1	0.73	0.6	0.64	0.49
ILMN_1700337	TROAP	0.73	0.86	0.77	0.69
ILMN_2215061	ORC6	0.74	0.98	0.78	0.66
ILMN_1806040	TYMS	0.74	0.46	0.6	0.37
ILMN_16427	CCNA2	0.75	0.74	0.91	0.57
ILMN_1709294	CDCA8	0.75	0.78	0.96	0.54
ILMN_4880	DLGAP5	0.75	0.77	0.84	0.43
ILMN_1697998	MTFR2	0.75	0.85	0.9	0.55
ILMN_1750508	STMN1	0.75	0.65	0.61	0.34
ILMN_1709484	BLM	0.76	0.7	0.75	0.43
ILMN_1716279	CENPE	0.76	0.69	0.61	0.57
ILMN_7092	EXO1	0.76	0.7	0.85	0.42
ILMN_1771039	GTSE1	0.76	0.71	0.89	0.44
ILMN_3057	CDCA5	0.77	0.78	0.81	0.56
ILMN_1753063	KIF15	0.77	0.75	0.72	0.43
ILMN_1731184	MELK	0.77	0.9	0.94	0.57
ILMN_1747016	CEP55	0.78	0.69	0.95	0.61
ILMN_20921	ANLN	0.79	0.76	0.83	0.48
ILMN_1815184	ASPM	0.79	0.63	0.94	0.42
ILMN_16725	DEPDC1B	0.79	0.75	0.78	0.55
ILMN_1799667	KIF4A	0.8	0.81	0.83	0.43
ILMN_1657600	NCAPH	0.81	0.73	0.7	0.53
ILMN_12357	UBE2C	0.81	1.13	0.97	0.76
ILMN_8354	CDC20	0.82	0.82	1.13	0.66
ILMN_1651237	CDT1	0.82	0.85	0.73	0.39
ILMN_137805	AURKB	0.83	0.91	0.92	0.72
ILMN_1781943	FAM83D	0.83	0.95	0.98	0.68
ILMN_8595	KIFC1	0.83	0.68	0.74	0.46
ILMN_24472	TTK	0.83	0.85	0.93	0.53
ILMN_1736090	BUB1	0.84	0.74	0.93	0.51
ILMN_1733396	CDC25A	0.84	0.94	0.84	0.54
ILMN_137041	HJURP	0.84	0.78	0.78	0.52
ILMN_1792494	TPX2	0.84	0.73	0.93	0.59
ILMN_1710082	BIRC5	0.85	0.93	1.04	0.77
ILMN_27839	KIF18A	0.85	0.76	0.72	0.51
ILMN_1664511	NDC80	0.85	0.65	0.74	0.57
ILMN_1730825	SGOL1	0.85	0.81	0.92	0.65
ILMN_1771224	SKA1	0.85	0.84	0.88	0.65
ILMN_25582	DEPDC1	0.87	0.77	0.95	0.5
ILMN_25969	KIF2C	0.87	0.78	0.99	0.65
ILMN_1727459	ORC1	0.88	0.76	0.91	0.43

ILMN_1751776	CKAP2L	0.89	0.69	0.77	0.5
ILMN_1706194	MCM10	0.9	1.02	1.01	0.66
ILMN_2392472	CENPA	0.91	0.89	1.07	0.68
ILMN_18637	RRM2	0.92	0.78	0.73	0.36
ILMN_29093	MYBL2	0.94	1.27	1.14	0.82
ILMN_1757697	NEIL3	0.94	0.85	0.75	0.53
ILMN_18676	FAM64A	0.95	0.79	1	0.5
ILMN_10352	FOXM1	0.95	0.83	0.99	0.57
ILMN_1808347	CDC45	0.96	0.87	0.95	0.6
ILMN_1753422	E2F8	0.96	0.92	0.92	0.71
ILMN_14100	KIF20A	0.96	0.81	0.82	0.49

Table S4. Correlation between stem cell-related transcription factors and ESR1, PGR, HER2 status.

Protein A	Protein B	Correlation	p value
CTNNB1	ESR1	0.10297	0.0023038
CTNNB1	PGR	-0.059108	0.08073
CTNNB1	HER2	0.30128	8.5142e-20
FOXM1	ESR1	No matching records found	
FOXM1	PGR	-0.2664	1.1515e-15
FOXM1	HER2	-0.093679	0.005578
FOXO3A	ESR1	No matching records found	
FOXO3A	PGR	-0.060053	0.076
FOXO3A	HER2	-0.11294	0.00082377
GLI2	ESR1, PGR, or HER2	No matching records found	
HIF1A	ESR1, PGR, or HER2	No matching records found	
HMGA1B	ESR1, PGR, or HER2	No matching records found	
KLF4	ESR1, PGR, or HER2	No matching records found	
MAF	ESR1, PGR, or HER2	No matching records found	
MCM2	ESR1, PGR, or HER2	No matching records found	
NANOG	ESR1, PGR, or HER2	No matching records found	
POU5F1	ESR1, PGR, or HER2	No matching records found	
PRDM14	ESR1, PGR, or HER2	No matching records found	
SNAI1	ESR1, PGR, or HER2	No matching records found	
SOX2/SOX9	ESR1, PGR, or HER2	No matching records found	
STAT3	ESR1, PGR, or HER2	No matching records found	
WWTR1	ESR1, PGR, or HER2	No matching records found	
TBX3	ESR1, PGR, or HER2	No matching records found	
TWIST1	ESR1, PGR, or HER2	No matching records found	
ZEB1	ESR1, PFR, or HER2	No matching records found	
LIN28 A/B	ESR1, PFR, or HER2	No matching records found	
MYC	ESR1	-0.15839	0.0000025322
MYC	PGR	0.032339	0.33961
MYC	HER2	-0.13336	0.000076665

Note: Data analysis was done on a dataset of TCPA Breast Invasive Carcinoma with 901 samples where RPPA data were available (http://tcpaportal.org/tcpa). P-value is based on Spearman's rank test. 'No matching records found' indicates that RPPA data for the protein in question is not available in the database for the test. ESR1: estrogen receptor subunit 1. PGR: progesterone receptor. HER2: human epidermal growth factor receptor 2.
